# Orchid–pollinator interactions and potential vulnerability to biological invasion

**DOI:** 10.1093/aobpla/plv099

**Published:** 2015-08-18

**Authors:** Adam D. Chupp, Loretta L. Battaglia, Eric M. Schauber, Sedonia D. Sipes

**Affiliations:** 1 Department of Plant Biology, Southern Illinois University, Carbondale, IL 62901, USA; 2 Department of Zoology, Southern Illinois University, Carbondale, IL 62901, USA

**Keywords:** Biological invasion, laurel wilt disease, nectar spur length, orchid pollination, *Papilio palamedes*, *Platanthera ciliaris*, pollinator availability, proboscis length

## Abstract

Mutualistic relationships between plants and their pollinators have played a major role in the evolution of biodiversity. While the vulnerability of these relationships to environmental change is a major concern, studies often lack a framework for predicting impacts from emerging threats (e.g. biological invasions). The objective of this study was to determine the reliance of *Platanthera ciliaris* (orange-fringed orchid) on *Papilio palamedes* (Palamedes swallowtail butterfly) for pollination and the relative availability of alternative pollinators. Recent declines of *P. palamedes* larval host plants due to laurel wilt disease (LWD) could endanger *P. ciliaris* populations that rely heavily on this butterfly for pollination. We monitored pollinator visitation and fruit set and measured nectar spur lengths of *P. ciliaris* flowers and proboscis lengths of its floral visitors in Jackson County, MS, USA. *Papilio palamedes* was the primary visitor with minimal visitation by *Phoebis sennae* (cloudless sulfur butterfly). Lengths of *P. ciliaris* nectar spurs were similar to proboscis lengths of both pollinator species. Fruit set was moderate with access to pollinators (55 ± 10.8 %), yet failed (0 %) when pollinators were excluded. Visitation increased with inflorescence size, but there was no such pattern in fruit set, indicating that fruit set was not limited by pollinator visitation within the range of visitation rates we observed. Our results are supported by historical data that suggest *P. palamedes* and *P. sennae* are important pollinators of *P. ciliaris*. Although *P. sennae* may provide supplemental pollination service, this is likely constrained by habitat preferences that do not always overlap with those of *P. cilaris*. Observed declines of *P. palamedes* due to LWD could severely limit the reproductive success and persistence of *P. ciliaris* and similar orchid species populations. This empirical-based prediction is among the first to document exotic forest pests and pathogens as an indirect threat to plant–pollinator interactions.

## Abstract (precis)

Plant reproduction is often limited by the availability of pollinators which are themselves dependent on larval host plants. The Palamedes swallowtail butterfly is abundant in the southeastern USA but is in decline due to the widespread mortality of its primary larval host plant. Our observations in the field and laboratory suggest that the orange-fringed orchid relies heavily on this butterfly for pollination and availability of alternative pollinators is low. We conclude that populations of this and similar orchid species are indirectly threatened by an exotic plant pathogen which kills the primary larval host of the Palamedes swallowtail.

## Introduction

It is estimated that 87.5 % of all flowering plants are pollinated by animals (Ollerton *et al*. 2011). The degree to which this service regulates plant reproductive success has been a popular subject of debate and research over the last several decades. Although Bateman's principle of sexual selection suggests that the reproductive output of female plants (seed set and maturation) is limited by resource availability rather than access to mates (pollen receipt) (Bateman 1948; Janzen 1977; Wilson *et al*. 1994), reviews of empirical data indicate that reproductive success is commonly (and often severely) limited by pollen/pollinator availability (Burd 1994; Ashman *et al*. 2004). It is clear that interactions between plants and their pollinators have played a major role in the evolution of biodiversity. Unfortunately, human activities have posed serious threats to the maintenance of these relationships.

The disruption of mutualistic relationships (e.g. plant–pollinator interactions) may lead to co-extinctions and a substantial decline in global plant diversity (Aslan *et al*. 2013; González-Varo *et al*. 2013). While several types of global environmental change have caused these disruptions (e.g. CO_2_ enrichment, nitrogen deposition, climate and land use), biological invasions have produced some of the most dramatic shifts in the composition of local and regional communities (Vitousek *et al*. 1996; Tylianakis *et al*. 2008). In North America, arguably the greatest threat to native communities is invasion by exotic phytophagous insects; there are now over 400 such species with at least one in nearly every forested habitat (Mattson *et al*. 1994, 2007). Among native species that are threatened by impacts from exotic insect herbivores, insect pollinators whose larval stages share a host species with the invaders are the most vulnerable (Gandhi and Herms 2010). Accordingly, the pollination services provided by the adult stages of these native insects are also threatened. Understanding how the disruption of these services may affect plant reproduction and persistence requires detailed analyses of plant–pollinator interactions.

Orchid species of the genus *Platanthera* are well studied and have been used as models for testing mechanisms of pollination and co-evolution. The orange-fringed orchid (*Platanthera ciliaris*) is a large, terrestrial orchid that has a patchy distribution across the eastern USA and Canada (USDA NRCS 2015). This orchid is most often associated with acidic, nutrient poor soils that are characteristic of pine flatwoods, savannahs and bogs. The orange flowers of *P. ciliaris* are apparently specialized for pollination by long-tongued insects (e.g. large butterflies) that make contact with pollinaria while retrieving nectar from the bottom of long nectar tubes (Smith and Snow 1976; Folsom 1984; Robertson and Wyatt 1990*a*, *b*). The pollinaria stick to the eyes of pollinators and are then brushed over stigmas on subsequent floral visits (Robertson and Wyatt 1990*b*). In mountain and coastal habitats of South Carolina, USA, *P. ciliaris* populations may exhibit ecotypic variation based on co-evolutionary relationships with the longest-tongued local pollinators, thus suggesting significant variability in pollinators’ identities and the morphological characteristics of plants and pollinators (e.g. proboscis and nectar spur lengths) across this region (Robertson and Wyatt 1990*a*). As with other plant species including North American orchids (*Spiranthes* spp.), breeding systems, floral morphology (e.g. nectar spurs), pollinators and pollinator morphology (e.g. proboscis length) may vary geographically across the entire range of an individual species (Catling 1982; Schmidt and Antlfinger 1992; Herlihy and Eckert 2005; Anderson and Johnson 2008). Thus far, the only analysis of *P. ciliaris* and its pollinator network was conducted in South Carolina nearly 30 years ago (i.e. Robertson and Wyatt 1990*a*, *b*), and more work is required to define the potential range of species that pollinate this orchid and the strength and variability of these relationships. In addition, given the high degree of specialization that has so far been identified between *P. ciliaris* and its pollinators, this orchid may be extremely vulnerable to the impacts of a recent widespread biological invasion.

In the southeastern USA, the Palamedes swallowtail (*Papilio palamedes*) is a common long-tongued butterfly and has been identified as a pollinator of *P.ciliaris*, but recent declines of its primary larval host (redbay tree [*Persea borbonia*]) due to laurel wilt disease (LWD) jeopardize the survival of this butterfly (Chupp and Battaglia 2014; J. P. Formby *et al.* in preparation). The fungal pathogen, which is vectored by an exotic ambrosia beetle (*Xyleborus glabratus*), is causing widespread mortality of redbay and other Lauraceae species and likely threatens the pollinator networks of *P. ciliaris* and many other plant species (Fraedrich *et al*. 2008; Mayfield 2008; Smith *et al*. 2009; Spiegel and Leege 2013; Evans *et al*. 2014; USDA Forest Service 2015). Abundance estimates of *P. palamedes* based on total field counts along transects in LWD-impacted areas are lower than estimates from non-impacted areas (Formby *et al*. in preparation). Thus, *P. palamedes* populations may already be declining in areas where LWD began to invade in 2009. While a range of Lepidopteran species are imperilled by the loss of their larval hosts due to other exotic insects such as the emerald ash borer (*Agrilus planipennis*), gypsy moth (*Lymantria dispar*), balsam woolly adelgid (*Adelges piceae*) and cottony cushion scale (*Icerya purchase*) (Work and McCullough 2000; Roque-Albelo *et al*. 2003; Scholtens and Wagner 2007; Wagner 2007), predictions of how these losses may affect the plants they pollinate are absent from the literature. Predicting these impacts requires foresight and detailed observations of relationships prior to disturbance.

Here, our focus is to identify the potential pollinators of *P. ciliaris* and predict the vulnerability of these mutualistic relationships. While historical data exist for this orchid (i.e. Robertson and Wyatt 1990*a*, *b*), it is unclear how the visitation rates and morphology of plants and pollinators may vary across time and the physiographic sections encompassed by its widespread but patchy distribution. While the primary objective of this study was to quantify temporal and spatial variability of a model plant–pollinator network, we provide a novel framework by suggesting vulnerability to imminent biological invasion. Despite the limited geographic scope of this study due to the already widespread impacts of LWD, we present timely data from an unimpacted area on the northern Gulf Coast, USA. We accomplished our objectives by addressing the following questions: (i) What is the relative abundance of local pollinator species and which are the most frequent visitors of *P. ciliaris* flowers? (ii) What is the breeding system of *P. ciliaris* (i.e. is visitation necessary for successful pollination and fruit maturation?) and how does the rate of fruit set and maturation compare with populations from other regions and habitats? (iii) What is the within-population variability of pollinator and flower morphology (i.e. proboscis and nectar spur lengths) and how does variation in these features compare with populations from other regions and habitats? (iv) Do the answers to the above questions indicate vulnerability to the impacts of LWD?

## Methods

### Study site

The study site was located on the Grand Bay National Estuarine Research Reserve (GBNERR) in Jackson County, MS, USA. In August 2012, we identified a population of *P. ciliaris* in an area of wet pine flatwoods that was surrounded on all sides by bald cypress-dominated (*Taxodium distichum*) wetlands. The pine flatwood vegetation consisted of a sparse canopy of slash and long-leaf pine (*Pinus elliottii* and *P. palustris*) and a diverse herbaceous understorey dominated by wiregrass (*Aristida stricta*). In addition to *P. ciliaris*, the site also contained a large population of the orange fringeless orchid (*Platanthera integra*), which is considerably smaller in stature compared with *P. ciliaris*. Due to fire suppression, several woody species (e.g. *Smilax laurifolia*, *Ilex glabra* and *Hypericum* spp.) were also encroaching into this area.

### Flower visitation

At our study site, *P. ciliaris* flowered for about a month from mid-August to mid-September; individual plants flowered for 2–3 weeks. Preliminary observations were conducted as inflorescences began to flower and at that time, very few pollinators were observed visiting flowers. We decided to concentrate our survey during a period of peak flowering, when pollinator abundance began to increase substantially. Following Robertson and Wyatt (1990*a*, *b*) who noted that nectar volume peaked between 4 and 7 days after anthesis, we determined a 3-day period in which plants contained the greatest number of flowers that were between 4 and 7 days post anthesis. In this way, we maximized survey time for the period in which pollinator attraction to flowers was greatest (i.e. peak flowering).

On 24–26 August 2012 (near peak flowering for the population), we recorded insect visitation to a total of 24 *P. ciliaris* plants. We recorded the number of open flowers on each plant (proxy for inflorescence size), tagged each plant with PVC pipe within 20 cm of plant and recorded their GPS coordinates. Plants were at least 0.5 m apart. All observation sessions were conducted between 08:00 and 16:00 h, the period of visitor activity, as indicated by preliminary observations. During the survey period, the weather remained consistent with daily high temperatures of 28–31 °C and partly cloudy skies with no rain (NOAA 2013).

We divided the observation plants into four groups in which individuals occurred in sufficiently close proximity to be observed simultaneously by one observer. Plants within a group were observed for a session lasting 30 or 60 min, after which the observer rotated to a different group. Because visits were very infrequent within some groups, and because we were interested in the relative, rather than absolute, frequency of the pollinator species, we focussed our survey on two of the four groups (*n* = 7 and 8 plants) with higher visitation.

The observer sat within 6 m of the grouping of plants being monitored. A visit was recorded when an insect arrived at a plant and inserted its proboscis in the nectar tube of at least one flower. Each time a visitor arrived at a plant, we recorded the identity of the visitor and the total number of flowers probed. Each arrival to a plant was treated as a visit (therefore, we do not know how frequently the same individual insect flew out of sight and later revisited the same plant). We calculated the visits per plant per hour as well as the mean number and proportion of open flowers probed per visit for each species of visitor. We were not able to record the total number of visits to individual flowers.

### Breeding system

To verify the importance of insect visitation for successful pollination and fruit set, we compared fruit set of inflorescences that were either bagged or open to pollinators. We excluded potential pollinators from five plants not included in the visitation surveys by placing lightweight mesh bags (1 mm) over inflorescences of unopened flowers. The mesh bags were left on until all flowers had completely dried (roughly 10 days after the end of our survey), at which point we collected the inflorescences of all bagged specimens. At the same time, dried inflorescences were collected from five of the plants used in the visitation observations (as open-pollinated controls). All specimens were placed in paper bags and kept in a drying oven at 50 °C.

**Figure 1.**
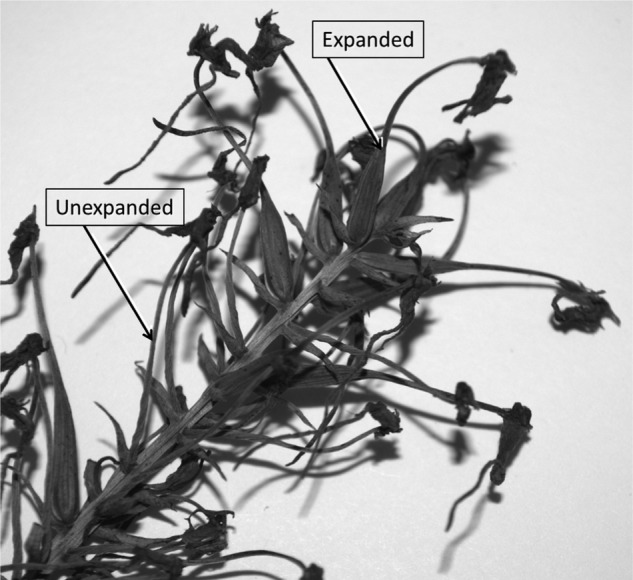
Expanded and unexpanded ovaries on a dried *P. ciliaris* inflorescence. The swelling of ovaries indicated fruit maturation which was verified through examination of dissected seeds (Fig. 2).

**Figure 2.**
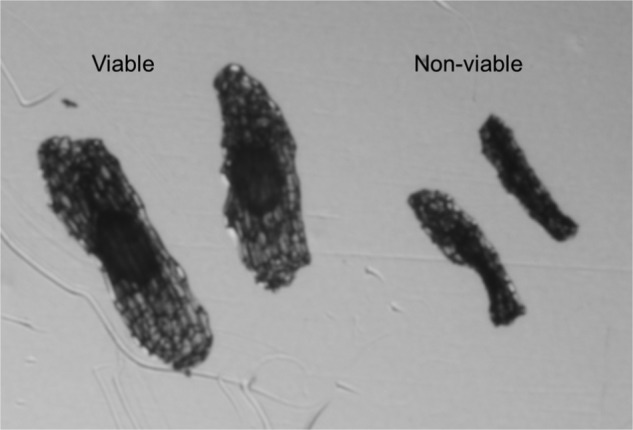
Viable and non-viable seeds that were dissected from expanded and unexpanded *P. ciliaris* ovaries, respectively. Viable seeds contain enlarged embryos in the centre of the seed. Viewed at ×90 magnification.

Successful pollination and fruit set were indicated by a widening of the ovary (Fig. 1). To ensure that we were accurately recognizing ovaries with viable fruits, we dissected a small subset of ovaries (*n* = 8) and examined the seeds under a dissecting microscope (Model SZX12, Olympus, Center Valley, PA, USA) to verify viability (i.e. embryonic enlargement, Fig. 2). Unexpanded ovaries always contained seeds with undeveloped embryos, while expanded ovaries consistently harboured seeds with developing embryos. In cases when ovaries exhibited moderate widening, seed viability was assessed under the microscope by examining the relative size of the embryo. For each inflorescence, fruit set was quantified as the proportion of flowers that had expanded ovaries (containing at least some viable seeds).

### Nectar spur and proboscis length

We collected two fully opened flowers from each of 22 randomly selected *P. ciliaris* plants. Flowers were placed in a 40 % ethanol solution and returned to the laboratory for measurement. Each flower was removed from the ethanol solution and pinned to Styrofoam just prior to measuring. The pinning allowed us to effectively isolate the nectar spur and accurately measure its length from the apex to its junction with the expanded portion of the labellum (Robertson and Wyatt 1990*a*).

Upon completion of our visitation surveys, we also collected individuals of the pollinator species that visited *P. ciliaris* flowers. Individuals were captured in the field and immediately taken to the laboratory where they were frozen. For each species, an equal number of females and males was collected (*n* = 10 of each sex for two species, 40 total). The specimens were later removed from the freezer and allowed to thaw before heads were amputated. Removed heads were pinned to Styrofoam and each proboscis was unrolled and carefully held in place with pins and small strips of paper. Proboscis length was measured from the apex to its junction with the labrum (Robertson and Wyatt 1990*a*).

### Statistical analyses

To analyse visitation by multiple visiting insect species, we considered the individual plants that we monitored to be our sample units. To test for differences in the number of visits per plant made by each pollinator species, we used a paired samples *t*-test. To calculate the mean number of pollinator visits per plant per hour, we pooled data for each plant across the total survey period. We used linear regression analysis to determine whether there was a relationship between the number of visits a plant received and (i) the number of plants being observed in that group and (ii) the number of open flowers on that plant. To test whether the number of flowers probed per visit differed between pollinator species, we used an independent samples *t*-test with individual visit as our sample unit. This same method of analysis was used to test for differences in the proportion of open flowers probed per visit between visiting species. For all *t*-tests, when the assumption of equality of variance was violated, we used results from the Satterthwaite approximation. Linear regression analysis was used to determine whether there was a relationship between the total number of flowers per inflorescence and the proportion of flowers that were successfully pollinated and set fruit; only results from unbagged plants were included in this analysis. One-way ANOVA and Tukey's *post hoc* test (where warranted) were used to test for differences among the lengths of pollinator proboscises and nectar spurs. Variances of nectar spur and proboscises lengths were compared using homogeneity of variance tests (Levene's). For each pollinator species, we also tested for differences in proboscis lengths between males and females using independent samples *t*-tests. Square-root transformations were applied to any data that did not meet normality and equality of variance assumptions. All statistical procedures were conducted using the SAS 9.3 software package (SAS Institute 2011).

## Results

### Flower visitation

**Figure 3.**
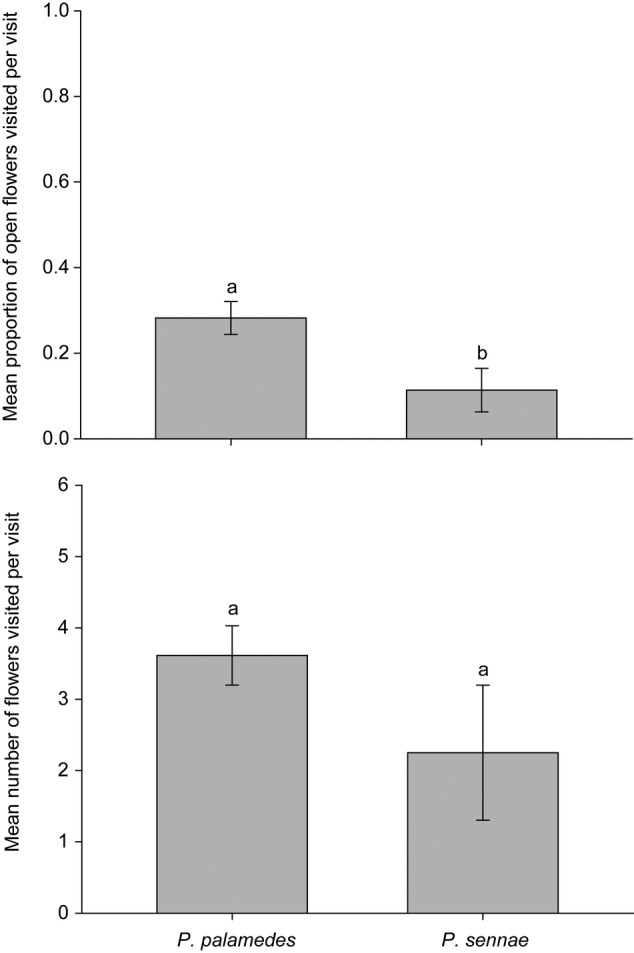
Mean proportion of open flowers visited per visit (top) and mean number of flowers visited per visit (bottom) by *P. palamedes* (44 visits) and *P. sennae* (4 visits). Different letters indicate significant differences between species (*P* < 0.05).

During our 3-day survey period, 11 total hours of observation time were recorded. Visitors were observed on 15 of the 24 plants that we monitored (48 visits total). *Papilio palamedes* (*n* = 44 visits) and *Phoebis sennae*, cloudless sulfur (*n* = 4 visits), were the only two species of visitor observed during this period (Table 1). The average number of visits plant^−1^ h^−1^ (±SE) was higher for *P. palamedes* (0.53 ± 0.12) than for *P. sennae* (0.03 ± 0.02) (*t* = 4.53, df = 23, *P* < 0.001). The mean number of flowers visited per visit was similar between *P. palamedes* (3.61 ± 0.42) and *P. sennae* (2.25 ± 0.95) (*t* = 1.32, df = 4, *P* = 0.26). The mean proportion of open flowers visited per visit was significantly higher for *P. palamedes* visits (0.28 ± 0.04) compared with *P. sennae* (0.11 ± 0.05) (Satterthwaite: *t* = 2.65, df = 7.3, *P* = 0.032) (Fig. 3). There was no apparent relationship between plant visitation (total number of visits to a given plant) and the number of plants in its group (*r*^2^ = 0.02, *F*_1,22_ = 0.48, *P* = 0.49). However, there was a marginally significant relationship (positive) between plant visitation and the number of open flowers on individual plants (*r*^2^ = 0.16, *F*_1,22_ = 4.21, *P* = 0.05).

**Table 1.** Pollinator activity on *P. ciliaris*. Visits are the number of times an individual of that species was observed nectaring on the flowers of individual plants. *Papilio palamedes* and *P. sennae* accounted for 44 and 4 visits, respectively. Mean ± SE values are given in the last row of the table.

Group	Plant ID	Number of flowers	Observation time (h)	Number of visits		Total visits	Visits plant^−1^ h^−1^
				*P. palamedes*	*P. sennae*		
1	1	19	5	9	1	10	2
1	2	22	5	8	1	9	1.8
1	3	9	5	1	0	1	0.2
1	4	20	5	5	2	7	1.4
1	5	21	5	2	0	2	0.4
1	6	5	5	2	0	2	0.4
1	7	5	5	0	0	0	0
2	8	20	2	0	0	0	0
2	9	9	2	2	0	2	1
2	10	13	2	0	0	0	0
2	11	7	2	0	0	0	0
2	12	7	2	0	0	0	0
2	13	14	2	0	0	0	0
2	14	9	2	0	0	0	0
3	15	9	1.5	1	0	1	0.7
3	16	20	1.5	0	0	0	0
4	17	10	2.5	1	0	1	0.4
4	18	10	2.5	3	0	3	1.2
4	19	5	2.5	2	0	2	0.8
4	20	9	2.5	1	0	1	0.4
4	21	11	2.5	1	0	1	0.4
4	22	12	2.5	2	0	2	0.8
4	23	14	2.5	4	0	4	1.6
4	24	5	2.5	0	0	0	0
		11.9 ± 1.2	3.0 ± 0.3	1.8 ± 0.5	0.17 ± 0.10	2.0 ± 0.6	0.6 ± 0.13

### Breeding system

The average number of flowers (±SE) on each inflorescence was 15.6 ± 3.6 on bagged specimens and 29.0 ± 4.4 on unbagged specimens. On bagged specimens, successful fruit set did not occur on any of the 78 flowers. However, on unbagged specimens, an average of 55 % (±10.8) of flowers had successfully set fruit. Results from a regression analysis indicated that there was no relationship between the total number of flowers on an unbagged inflorescence and the proportion that set fruit (*r*^2^ = 0.01, *F*_1,3_ = 0.03, *P* = 0.87).

### Nectar spur and proboscis length

**Figure 4.**
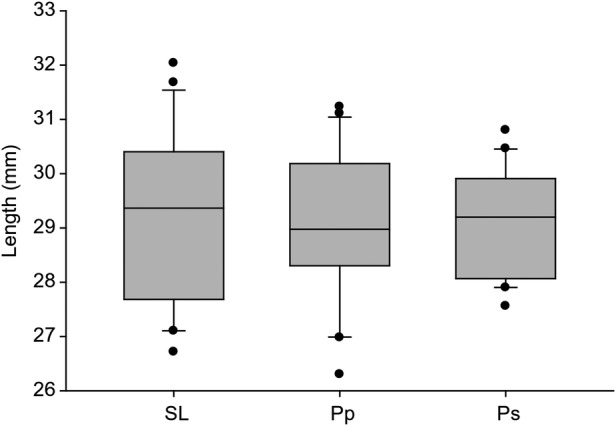
Relationship between nectar spur length of *P. ciliaris* (SL) and the proboscis lengths of *P. palamedes* (Pp) and *P. sennae* (Ps). The horizontal line is the median and the boxes and error bars represent the 10th, 25th, 75th and 90th percentiles. Black dots are outliers. There were no significant differences (*P* > 0.05).

Average spur length (±SE) estimated from 44 flowers (22 plants) was 29.10 ± 0.33 mm. *Papilio palamedes* and *P. sennae* were the only two species of visitor observed during our survey period, and thus, we measured proboscis length on these two species only. Average proboscis lengths of *P. palamedes* and *P. sennae* were 29.06 ± 0.30 and 29.12 ± 0.22 mm, respectively. Results of ANOVA suggested no significant differences in lengths among proboscises of *P. palamedes* and *P. sennae* and spurs of *P. ciliaris* (*F*_2,59_ = 0.01, *P* = 0.99) (Fig. 4). There was no influence of sex on proboscis length in *P. palamedes* (male: 29.58 ± 0.32 mm, female: 28.55 ± 0.47 mm) (*t* = 1.85, df = 18, *P* = 0.08) or *P. sennae* (male: 28.97 ± 0.27 mm, female: 29.27 ± 0.36 mm) (*t* = 0.69, df = 18, *P* = 0.50). The variance of nectar spur lengths did not differ from that of *P. palamedes* proboscis lengths (*F*_20,19_ = 1.37, *P* > 0.05). However, variance of *P. sennae* proboscis lengths was lower than for nectar spur lengths (*F*_20,19_ = 2.54, *P* < 0.05).

## Discussion

Results of our survey suggest that only two species, *P. palamedes* and *P. sennae*, are visiting and potentially pollinating the flowers of *P. ciliaris* in our study area; these were also the only two species observed during preliminary surveys. Although the identity of these primary visitors is consistent with observations from the Atlantic Coastal Plain (Robertson and Wyatt 1990*a*), the proportion of visits by each species differed substantially. *Papilio palamedes* represented 92 % of our observations while it accounted for only 63 % of visits (2-year average) in the surveys conducted by Robertson and Wyatt (1990*a*); despite inter-annual variation in the total number of individuals they observed, the proportion of visits made by *P. palamedes* and *P. sennae* was consistent between years (Robertson and Wyatt 1990*a*). Our results indicate that *P. ciliaris* populations in the area of our study site rely heavily on *P. palamedes* for floral visitation and that LWD-induced declines of *P. palamedes* threaten the reproduction and persistence of this orchid species.

Overall, we found that an alternative pollinator of *P. ciliaris*, *P. sennae*, was much less abundant than implied by the observations of Robertson and Wyatt (1990*a*). *Phoebis sennae* prefers edges and open areas, while *P. palamedes* is more closely associated with forested habitats (e.g. Devries 1987; Haddad 1999; Haddad and Baum 1999). At the site of our surveys, the sparse pine canopy and often thick understorey layer may be less suitable for *P. sennae* than other more open and/or disturbed areas. The median of a nearby highway (I-10) contained very high densities of *P. sennae*, presumably attracting individuals away from less favourable neighbouring habitats (A. D. Chupp, pers. obs.). In addition, the availability of larval host plants (*Cassia* spp.) influences habitat suitability and temporal fluctuations in the local abundance of *P. sennae* may have also been a factor during our survey period.

We maintain that long-tongued pollinators (i.e. *P. palamedes* and *P. sennae*) are the only floral visitors and pollinators of *P. ciliaris* due to the retaining of nectar in the bottom of long nectar spurs; shorter-tongued pollinators are unable to reach the nectar and are thus not attracted to these flowers. While visitation by *P. sennae* was minimal, *P. palamedes* visited 62 % of the plants we monitored. As pollinator exclusion bags resulted in 0 % fruit set on bagged inflorescences, we conclude that visitation by *P. palamedes* was primarily responsible for pollination and fruit set. This result is consistent with previous findings which confirmed that *P. palamedes* carried significantly more pollinaria than *P. sennae* (Robertson and Wyatt 1990*b*). However, among unbagged inflorescences, only 55.2 % of flowers set fruit and variability among plants was high (±10.8 % SE). On the Coastal Plain of South Carolina, variability in fruit set was explained by differences between the lengths of *P. cilaris* nectar spurs and pollinator proboscises whereby greater similarity was correlated with higher rates of pollination success and fruit set (Robertson and Wyatt 1990*a*). Here, we report only moderate fruit set in *P. ciliaris* despite results that indicate the average lengths of individual nectar spurs and pollinator proboscises are well matched.

If nectar spur lengths are optimal for ensuring pollination, then it remains unclear why these results suggest a lower rate of fruit set than what has been observed in other populations (Robertson and Wyatt 1990*a*). We point out that proboscis length of *P. palamedes* ranged from 26.3 to 31.2 mm and males tended to have longer proboscises (29.6 ± 1.0 mm) than females (28.5 ± 1.5 mm). Such discrepancy could explain lower fruit set if males visited flowers more frequently than females and were able to rob nectar without making contact with pollinia; females with shorter proboscises would have to probe deeper to reach nectar and would, therefore, be more likely to make contact with pollinia. Although we were unable to document the sex of individual visitors, our sampling of *P. palamedes* and *P. sennae* populations indicated that males were indeed more abundant or at least more likely to be captured near our site. Documentation of pollinator sex ratios is not common, but it has been shown that male *P. helenus* and *P. protenor* visit the flowers of *Clerodendron trichotomum* more frequently than females (Suzuki *et al*. 1987).

Alternatively, if the floral visitors of *P. ciliaris* are providing efficient pollen delivery, resource limitation could then explain variability in fruit set and why plants with larger inflorescences (i.e. more open flowers) attracted more visitors but did not produce a greater number of fruits than plants with smaller inflorescences. We note that the average inflorescence size as dictated by the number of flowers per plant at our site (11.9 ± 1.2) is at the low end of what has been documented for this species (10–50 per plant) (Smith and Snow 1976; Folsom 1984). In *Platanthera bifolia*, fertilizer treatments increased capsule production in plants with smaller inflorescences, indicating poorer nutrient stores in these individuals (Mattila and Kuitunen 2000). As with differences in the abundance of *P. sennae* between this study and that of Robertson and Wyatt (1990*b*), we suggest that biotopic or microhabitat differences are responsible for the smaller inflorescences and reduced fruit set reported here. Resource availability (i.e. light and nutrients) at our survey site may be increasingly threatened by competition with woody species that are invading the understorey layer. Unfortunately, the fires that naturally maintained these habitats have been suppressed, and prescribed burning at the GBNERR is limited by the complexity of land ownership and resultant need for increased personnel and funding for burns (W. Underwood, pers. comm.). Successful conservation will require careful analyses of the local factors that pose immediate threats to these communities and timely intervention.

## Conclusions

Our results are drawn from only one study area, highlighting the general paucity of information about pollinator populations and communities, but they concord with published work from other sites implicating *P. palamedes* in pollination of *P. ciliaris*. Although the availability of abiotic resources and pollinators (specifically *P. palamedes*) may interact to determine the fitness of *P. ciliaris* and the maintenance of populations, we predict a marked decline in the reproductive success of *P. cilaris* plants following LWD. Expected LWD-induced declines of *P. palamedes*, whose larvae primarily feed on redbay (Brooks 1962; Scriber *et al*. 1991, 2008; Lederhouse *et al*. 1992), may dramatically reduce pollination service to *P. ciliaris* populations. This prediction is based on the results of our surveys which identify *P. palamedes* as the primary pollinator of *P. ciliaris* and the assumption that recent observations of *P. palamedes* declines will continue as the impacts of LWD spread. As an abundant pollinator, *P. palamedes* may also serve as the primary pollinator of other co-occurring native plants, including the white-fringed orchid (*Platanthera blephariglottis*) that also harbours nectar in exceptionally long nectar spurs (Smith and Snow 1976). To prepare for declines in *P. palamedes*, we advise land managers to implement strategies that could increase habitat suitability for other long-tongued pollinators that visit these orchid species (i.e. *P. sennae*) and for the orchids themselves. Such efforts include understorey clearing/burning and the creation of corridors between suitable habitats. We urge conservation biologists/ecologists, land managers and administrators to consider the effects that exotic forest pests and pathogens may have on native insect herbivores and the plants they pollinate.

## Sources of Funding

This research was conducted in the National Estuarine Reserve System under an award from the Estuarine Reserves Division, Office of Ocean and Coastal Resource Management, National Ocean Service, National Oceanic and Atmospheric Administration (#NA11NOS4200080).

## Contributions by the Authors

All authors have seen and agreed to the submitted manuscript. The lead author co-developed this study, conducted all the research, analysed the data and wrote the initial manuscript. All other authors assisted with developing the project and research design, edited the manuscript and helped prepare the paper for submission.

## Conflict of Interest Statement

None declared.
